# Future directions for reducing inequity and maximising impact of child health strategies

**DOI:** 10.1136/bmj.k2684

**Published:** 2018-07-30

**Authors:** Sarah L Dalglish, Joanna J Vogel, Geneviève Begkoyian, Luis Huicho, Elizabeth Mason, Elisabeth Dowling Root, Joanna Schellenberg, Abiy Seifu Estifanos, Rajani Ved, Fernando C Wehrmeister, Guilhem Labadie, Cesar G Victora

**Affiliations:** 1 Department of Maternal, Newborn, Child, and Adolescent Health, World Health Organization, Geneva, Switzerland; 2 Unicef, Beirut, Lebanon; 3 Centro de Investigación en Salud Materna e Infantil, Centro de Investigación para el Desarrollo Integral y Sostenible and School of Medicine, Universidad Peruana Cayetano Heredia Lima, Peru; 4 Institute for Global Health, University College, London, UK; 5 Department of Geography and Division of Epidemiology, Ohio State University, Columbus, Ohio, USA; 6 Department of Disease Control, London School of Hygiene and Tropical Medicine, London, UK; 7 School of Public Health, College of Health Sciences, Addis Ababa University, Addis Ababa, Ethiopia; 8 National Health Systems Resource Center, New Delhi, India; 9 International Center for Equity in Health, Postgraduate Program in Epidemiology, Universidade Federal de Pelotas, Pelotas, Brazil

## Abstract

Current global child health strategies have reduced wealth based inequities in care seeking for childhood illness, but we need much greater emphasis on equity in strategy design and implementation, say **Sarah L Dalglish and colleagues**

**Key messages**

Equity oriented child health policies are cost effective, improve coverage faster, and result in greater gains in child health
Global strategies have contributed to reducing inequities in child health over the past 20 years
Greater emphasis on equity in strategies’ design and implementation could have led to greater improvements
Future strategies must emphasise intersectoral action to tackle social determinants, targeted coverage of interventions, and sustainable financing to support poor families
Top-down leadership and bottom-up demand are needed to drive policy changes that benefit marginalised populations

Over the past two decades, the world has made considerable progress in reducing under 5 mortality, but not all children have benefitted, and stark inequities in coverage of interventions persist in nearly all countries.^1^ ^2^ Integrated Management of Childhood Illness (IMCI) was designed by Unicef and the World Health Organization to reach all children in countries with under 5 mortality rates greater than 40 per 1000 live births and has been implemented in over 100 countries since the mid-1990s. In 2012 WHO and Unicef introduced integrated Community Case Management (iCCM) as a complementary strategy to IMCI to extend case management to children living in underserved areas (box 1). Although IMCI lacks an explicit mechanism to reach children unable to access health facilities owing to poverty, marginalisation, or lack of coverage, iCCM has a stated equity goal of reaching underserved children.^3^

Box 1 **What are IMCI and iCCM?**

*Integrated Management of Childhood Illness (IMCI)—*introduced by WHO and Unicef in the mid-1990s, aims to reduce death, illness, and disability and to promote improved growth and development among children under 5. IMCI includes both preventive and curative elements that are implemented by families and communities as well as by health facilities. The strategy includes three main components: improving case management skills of healthcare staff; strengthening health systems; and improving family and community health practices.

*Integrated Community Case Management (iCCM)—*introduced by Unicef and WHO in 2012 as an equity focused strategy to complement and extend the reach of public health services by providing timely and effective treatment of common illnesses to populations with limited access to facility based healthcare providers and especially to children under 5. Under iCCM, frontline workers in the community are trained, supplied, and supervised to diagnose and treat malaria, pneumonia, and diarrhoea in children with an integrated approach using artemisinin based combination therapies, oral antibiotics, oral rehydration salts, and zinc.

The 2016 strategic review of IMCI and iCCM assessed the past two decades of implementation and drew lessons for meeting future child health goals, including those related to equitable outcomes, under the UN’s sustainable development goals and the Global Strategy for Women’s, Children’s and Adolescents’ Health (2016-30).^4^ It drew on qualitative and quantitative data sources from over 90 countries, hundreds of experts in global child health, and reviews of the scientific literature.^5^ Based on this review, we assess the contribution of IMCI and iCCM to reducing child health inequities, focusing on wealth and place of residence, and discuss ways to promote equitable child health outcomes in future strategies. We also analyse data from Demographic and Health Surveys (DHS), the global IMCI and iCCM implementation survey, and other sources (see supplementary materials).

## Contribution of IMCI and iCCM to improving child health equity

Inequities in health are unjust, socially produced disparities in health resources or outcomes due to differences in characteristics such as socioeconomic status, level of education, place of residence, sex, age, and ethnicity.^6^ Although systematic inequities persist in access to child health interventions, equity gaps in care seeking and intervention coverage for childhood illness have fallen in recent years.^7^ The contribution of IMCI and iCCM to these reductions is difficult to assess given that the extent of implementation varied widely and is probably confounded by national governance, resource spending, and prioritisation of child health within countries. Previous reviews of IMCI, including the 2001-05 multicountry evaluation, found that it was often implemented to a lesser extent in disadvantaged areas owing to existing health systems challenges, so it did not tend to promote equity within countries.^8^ ^9^ ^10^ As for iCCM, implemented widely only after 2010, insufficient data exist on its direct impact on equity,^11^ but reviews indicate that insufficient use of health services owing to lack of awareness and suboptimal care seeking has limited its impact in many contexts.^12^ ^13^ ^14^

**Fig 1:**
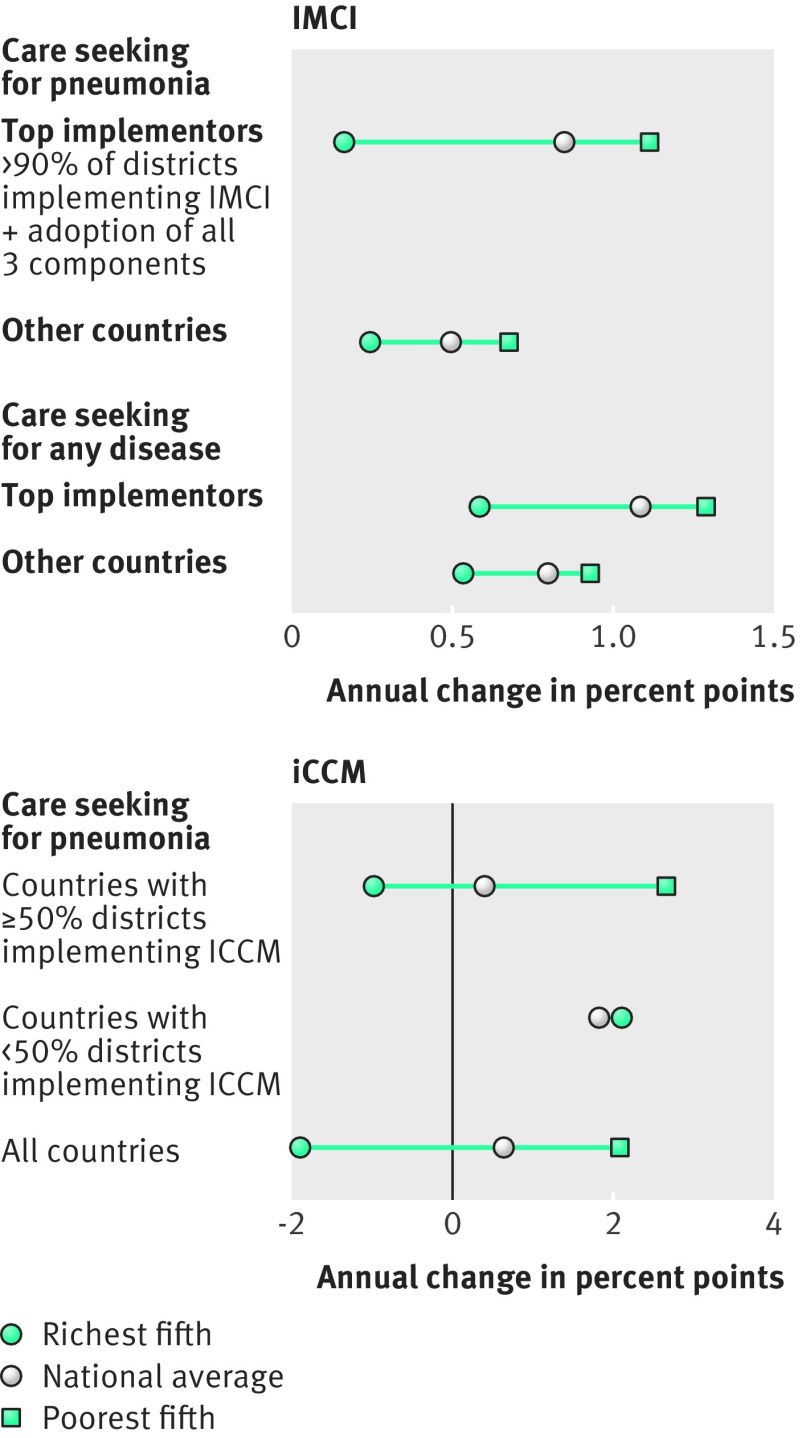
IMCI and iCCM implementation and annual percentage point increase in care seeking by wealth. Based on data from Demographic and Health Surveys (DHS) and the IMCI implementation survey. The analysis for IMCI is based on 90-147 surveys in 31-48 countries (1994-2010). The analysis for iCCM is based on 24-27 surveys in 18-22 countries (2010-14). For more detail on how these analyses were produced, see supplementary materials.

We tested the association between implementation of these strategies and reduction in wealth based inequities in care seeking for children with pneumonia or with any disease, using repeated panel analyses of DHS data (1993-2014) and IMCI and iCCM implementation data from a 2016 global survey from WHO and Unicef.^15^ We analysed IMCI and iCCM separately, as they were often implemented separately and over different time periods. The “top implementers” of IMCI (countries with >90% of districts implementing all three components) had notably faster annual increases in care seeking for pneumonia or for any disease, both as a national average and as a relative rise in the poorest fifth (fig 1) (based on 90-147 surveys in 31-48 countries; see supplementary materials). Because IMCI is a system-wide intervention with often partial implementation, fuller implementation to reach all children might have resulted in even greater increases in the poorest countries.

iCCM is a more recent strategy, implemented widely in sub-Saharan Africa beginning around 2010 and aiming to extend case management to underserved populations. Its effect on equity has been mixed (fig 1): countries with greater implementation (≥50% of districts implementing versus <50% of districts) had slower average annual growth in care seeking for pneumonia (0.4% point annual change versus 1.9%), slightly faster increases among the poorest fifth (2.7% point annual change versus 2.1%), and a notably greater spread across all countries (not enough DHS surveys were available to calculate care seeking for any disease; see supplementary materials). The contribution of iCCM to reducing inequities is complicated by the strategy’s recent advent and the lack of detailed data about the extent of district implementation. The greater benefit in the poorest fifth of higher implementing countries could be a result of the strategy’s focus on underserved children.

Both IMCI and iCCM seem to facilitate reductions in wealth based inequities in care seeking for childhood illness, but we think that emphasis on equity in strategy design and implementation could have led to greater improvements. We identified several blind spots in the design and implementation of these strategies that should be tackled to ensure maximal impact on inequities.

## Intersectoral action is needed to tackle social determinants of health

Intersectoral interventions are those undertaken by sectors such as education, environment, or finance, with or without the active collaboration of the health sector, to improve health outcomes. Over the past two decades, countries using intersectoral interventions to tackle the social determinants of child health were most successful compared with all other countries in reducing inequities and reducing child mortality rates overall.^16^ ^17^ Under the Countdown to 2015 initiative (which focused on the 75 countries with the greatest burden of maternal and child mortality), case studies of countries including Bangladesh, China, and Peru showed that they relied heavily on comprehensive improvements in water and sanitation, education of mothers, and poverty reduction programmes to sharply reduce inequities and overall under 5 mortality.^18^ ^19^ ^20^ ^21^

However, the strategic review found that, globally, countries rarely used IMCI or iCCM to tackle social determinants of health, and their designs did not emphasise intersectoral approaches. Both strategies were conceived primarily as medical interventions targeting childhood illness, with less focus on the child’s overall wellbeing or the social determinants of health. Although IMCI provides room for intersectoral activities under its third component (improving family and community practices, known as community IMCI or C-IMCI), this component was implemented to a lesser extent, owing in part to a lack of guidance, leadership, and financing from global partners. Of 94 countries that responded to the IMCI global implementation survey, 78 (83%) had conducted activities related to C-IMCI, whereas 92 (98%) had implemented the first component (health worker training). Country assessments indicated that C-IMCI activities were often scattershot, with no long term gains in programming. When iCCM was introduced, in part to bolster the ailing community component, it also focused on case management, not social determinants, reflecting a preference for medicalised solutions to population health problems.22

Global policy makers should prioritise intersectoral interventions, provide support for implementation, and articulate to national decision makers how these are complementary to IMCI and iCCM. WHO’s Health in All Policies framework, based on the notion that action in all sectors is required to improve the health of the poorest, is relevant but requires strong government ownership of child health goals and systematic coordination between sectors.^23^ In Peru, for example, government leadership and political will led to a move away from vertical programmes with an exclusive emphasis on maternal and child health, a decision that was key to the country’s success in reducing under 5 mortality.^21^ ^24^ Global and national stakeholders should boost political advocacy while also providing coordinated guidance and support for intersectoral activities, in line with the focus on children’s healthy development and the relation between health and all 17 of the UN’s sustainable development goals.

## Geographic coverage of services must be planned to match needs

Many health systems were underdeveloped when IMCI was introduced, and many countries selected districts for implementation based on feasibility rather than need. IMCI was implemented to a lesser extent in poorer or more remote districts in Peru and Tanzania, for example, often owing to poorer health systems in those areas. In Brazil, IMCI was less implemented in municipalities with low per capita income and small populations or those that were far from the state capital.^10^ iCCM brought case management services to more underserved areas from 2010, but the objective of improving population coverage of effective treatment interventions was not often achieved.^14^ Persistent geographic differences in coverage and health outcomes reflect an “urban bias” in service provision but can also result from longstanding neglect of poor populations, ethnic minorities, and indigenous populations.

**Fig 2:**
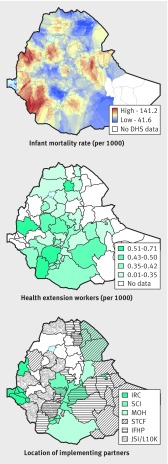
Mapping of child health needs and resource allocation in Ethiopia. Infant mortality was calculated from 2011 Demographic Health Survey (DHS) data and represents 10 year rates. Health extension worker and implementing partner data were collected as part of the strategic review and represent services in 2016. More details available in supplementary materials. IRC=International Rescue Committee; SCI=Save the Children International; MOH= ministry of health; STCF=Save the Children, Finland; IFHP=Integrated Family Health Program; JSI/L10K=John Snow Inc/Last 10 Kilometres Project.

Coverage of child health services is often incongruent with population needs. Stakeholders in Ethiopia, Nepal, and Yemen said that planning for IMCI implementation was not explicitly equity focused, resulting in a failure to target areas where mortality was higher and intervention coverage was lower. Many countries failed to coordinate activities between governmental and non-governmental actors and to target resources to areas of need. Maps of under 5 mortality in Ethiopia show regions in clear need of intervention, but health extension workers are not necessarily more numerous in these areas (fig 2). Ethiopia’s ministry was more successful in coordinating coverage than many other surveyed countries, but maps still show some areas served by multiple organisations and some by one (or none).

Programmatic tools are available to support the rational, equitable distribution of health services by geographic area, which policy-makers may find preferable to targeting by wealth quintile.^25^ Global positioning and geographic information systems can be used to identify areas of high mortality, low coverage, and suboptimal distribution of human resources. Geographic information applications can also be used to verify implementation, as in South Sudan, where the majority of community based health workers were deployed within a 5 km radius of a health facility or another health worker, contrary to programme planning and design.^26^ More rational planning for implementation will require global technical support to help countries prioritise and match investment with unmet needs. In Egypt, systematic planning for IMCI implementation focused first on districts with high under 5 mortality rates; three quarters of districts were covered in seven years, resulting in rapid declines in mortality.^27^ Systematic planning was enabled by strong political commitment, with the Egyptian government providing a dedicated budget and strong programme staff at national, governorate, and district levels, alongside coordinated support from partners. Similarly, new vaccines in Peru are rolled to the districts with highest mortality first, moving to other areas only after high coverage is reached.^28^

## Sustainable funding must reduce the burden on poor children’s families

Insufficient funding of child health programming is a cause of substandard public services, with the resulting burden of inferior care and out of pocket expenses falling disproportionately on poor families. The largest equity gaps affect interventions that require 24 hour access to health facilities.^1^ Elimination or reduction of user fees for child health services, recommended by WHO and shown to promote equitable outcomes by increasing service use most among the poorest,^29^ ^30^ is still not in force in many settings. In other contexts, elimination of service fees has not been conducive to the provision of high quality health services, because implementing the reform without preparing alternative funding mechanisms can increase access at the expense of quality.

Problems with sustainable financing are caused by more than just a lack of available funds. For the past two decades, the failure of partners to coordinate efforts globally and within countries was, in the words of one interviewed stakeholder, “inexcusable,” resulting in lopsided implementation, with some policy components and geographic areas receiving disproportionate resources and others going without. The Global Financing Facility—a World Bank initiative to coordinate resources from grants, governments, and private sources, bringing complementarity to partners’ support for maternal and child health—should be closely monitored for its effect on equity.

Lack of coordinated funding by global partners was compounded by insufficient allocation of funds. In the IMCI global survey, 60% of countries (55/85) cited the lack of a dedicated budget line in the health sector plan as a major barrier to implementation. “Cost of the programme” and “budget for training” were also cited as barriers at the regional level in 55% of countries (50/91) and 82% of countries (75/91), respectively. Meanwhile, only a minority of countries report plans to increase the proportion of funding for iCCM from domestic resources,^31^ possibly as these are often viewed as a “donor owned” strategies, with community health workers not being perceived as formal members of the health system.

Solutions are available to ensure adequate financing and improve the equitable distribution of resources for child health. National policy makers should understand and agree that policies targeted at equity, although more costly to implement, result in faster rises in coverage and sharper decreases in child mortality than non-targeted approaches and are cost effective in terms of cost per life saved.^7^ ^32^ Policy makers may be most convinced by analyses using data from their own countries, for example using equity focused monitoring and evaluation tools such as EQUIST (http://www.equist.info/en/dashboard), INNOV8 (http://www.who.int/life-course/partners/innov8/innov8-approach/en/), and the Health Equity Assessment Toolkit (http://www.who.int/gho/health_equity/assessment_toolkit/en/). These efforts could double as much needed evaluations establishing the tools’ differential applications or complementarity. Policy makers should also consider promoting health insurance to provide financial protection for families and reducing out of pocket spending by supporting transport costs through facilitated referral. Transferring money directly to beneficiaries has also been associated with better health outcomes for poor families,^33^ as in Mexico, where the Progresa programme showed the greatest reduction of diarrhoea incidence among children in the most deprived households.^34^ Some evidence indicates that these cash transfers can effectively mitigate monetary poverty and improve nutrition, school attendance, and use of health facilities.^35^

## Driving equity from the top**-**down and bottom**-**up

Equity oriented policies focus on the disproportionate burden of ill health among disadvantaged groups; on geographic, financial, and psychosocial barriers to access to services; and on remedial measures both inside and outside the health system.^36^ Many tools already exist: poverty reduction programmes and other intersectoral means of tackling social determinants of child health; geographic information systems and similar analytic tools to ensure equitable service delivery; and improved financing models. These approaches should be incorporated into IMCI and iCCM in coherent, complementary ways to maximise their equity promoting effects. Better data on health outcomes stratified by equity variables is sorely needed. Structured, comparative evaluations of programmes’ effects on health equity should be prioritised^37^ ^38^ so that national stakeholders can redress gaps in coverage, access, and outcomes, especially for the most vulnerable children.

To support such policies, countries need more resources. This will require government ownership and political will, to be spurred as necessary by well funded advocacy efforts. In our research, some countries reported advocacy or lobbying activities aimed at government, civil society, and industry (box 2), but we need to understand how to better engage these stakeholders. Many stakeholders interviewed for our research also said that IMCI could be transformed from a strategy to a programme, to encourage greater state financing and sustainable donor commitments. In any case, strong country planning must be backed by much more effective global coordination of resources, as has too rarely been the case over the past two decades.

Box 2 **Equity oriented strategies used by countries to increase access to and coverage of child health interventions**

*Intersectoral interventions*—follow-up of children in schools (Niger); intersectoral commission on early childhood development led by presidency (Colombia); Communication for Development approaches (multiple countries)
*Outreach to underserved populations*—Roving Caregivers Programme (Grenada); strengthening of outreach clinics (Nepal); outreach to children in remote communities (Guyana); Lady Health Worker Programme (Pakistan); mobile health teams in remote areas (Yemen)
*Social protection*—community based insurance (many countries); universal child health allowance (Argentina); integral health insurance (Bolivia); conditional cash transfers (Mexico)
*Rights based approaches*—incorporation of a rights based approach with consideration of cultural appropriateness (multiple countries, many in Latin America)
*Financing innovations—*removal of user fees (many countries); mobilisation of resources with the oil sector (Republic of the Congo); providing dedicated funding under the Health Development Fund (Zimbabwe); implementing results based funding (multiple countries); inclusion of a child health plan in the government’s national budget (Dominican Republic)
*Advocacy*—political lobbying to scale up IMCI (Mauritania); involving paediatric associations and civil society in promoting child health (Cameroon, Republic of the Congo)

Source: IMCI implementation survey

The underlying drivers of these changes must come from top-down leadership on equity and bottom-up demand for high quality services from children’s families and communities. National stakeholders in all countries have identified increased political commitment for IMCI as a priority, and reviews of iCCM have found that scaling up will require understanding and harnessing political accountability.^39^ Community engagement strategies can be a platform for building bottom-up accountability by sharing monitoring data and results, ideally in simple scorecard form, to serve as a basis for demanding service delivery.^40^

Equity oriented child health policies fulfil a basic human right; they are also cost effective, improve coverage faster, and result in greater gains in child health indicators. In the upcoming WHO and UNICEF initiative to redesign child health guidance and in implementing child health strategies worldwide, stakeholders should focus on ensuring equitable delivery of sustainably financed intersectoral services—with leadership and accountability to make it happen.

## Web extra

Extra material supplied by authors

Supplementary material on GIS development and mapping

Supplementary materials on IMCI and iCCM implementation analyses

Supplementary materials on care seeking for pneumonia or any disease

## Notes

See www.bmj.com/child-health for other articles in the series

## Notes

Contributors and sources: This article is based on findings from a global review of Integrated Management of Childhood Illness (IMCI) and integrated Community Case Management (iCCM) commissioned by the World Health Organisation. SLD conceived the outline of this paper and wrote the first draft; CGV, FCW, and GL performed the statistical analyses underpinning figure 1, and EDR performed the statistical analyses underpinning figure 2. All authors contributed intellectual content, edited the manuscript, and approved the final version for submission.

Provenance and peer review: This article is part of a series based on findings from a global review of Integrated Management of Childhood Illness funded by the Bill and Melinda Gates Foundation. Open access fees were funded by the Health Systems Research Unit, South African Medical Research Council. *The BMJ* peer reviewed, edited, and made the decision to publish the article with no involvement from the Medical Research Council.
