# Supplementary material for: Future directions for reducing inequity and maximising impact of child health strategies
Source: BMJ. 2018 Jul 30;362:k2684. doi: 10.1136/bmj.k2684 (PMC6283368; doi:10.1136/bmj.k2684)
Supplement: Supplementary file 1 — Supplementary material on GIS development and mapping [file dals042256.ww1.pdf]

Supplementary Material on GIS Development and Mapping

<u>Data</u>

Mortality Outcomes

The Demographic Health Surveys (DHS) are a population-representative household survey maintained by the DHS Program, implemented by ICF International, and funded by USAID (http://www.dhsprogram.com). The DHS have a variety of indicators on child morbidity and mortality, and most now collect Global Positioning System (GPS) data using GPS units which can be used to examine health indicators for small subnational areas. A 2014 report by USAID provides recommendations for geospatial analysis, suggests indicators appropriate for spatial analysis, and best practices for analyzing and displaying geographically explicit data [1]. This report supports geospatial analysis of DHS data and suggests it is reliable and accurate at the subnational level if analyzed correctly. At the same time, the DHS is a survey and subnational analyses can be subject to small numbers issues (especially for rare events such as infant mortality). In areas where sample sizes are small, rates and proportions should be interpreted with caution, and maps may reflect small numbers by showing areas with particularly high or low rates that do not accurately represent the reality of health outcomes in the area. In addition, some regions of a country may have no data at all. This occurs for a variety of reasons – e.g., populations are nomadic and difficult to sample, conflict creates dangerous operating conditions for interviewers.

DHS attempts to conduct surveys every 5-6 years in priority countries, but in some regions (especially those with significant conflict and political instability) the timing between surveys may be longer. Here we utilize the 2011 Ethiopia DHS. From the Standard DHS files, the birth recode and child recode files were used; these files are structured as one row per child with the potential of multiple children per eligible woman interviewed. We use the following specific indicators from the DHS data:
Infant Mortality and *Under 5 Mortality* were developed using the SAS *DHS_U5M* package [2] which utilizes the birth roster from DHS to calculate 5- or 10-year mortality rates. The DHS birth roster asks all eligible women in the household to list all live births, whether the child is currently alive or dead and, if the child died, the date of death. This information is used to produce estimates of the infant mortality and under 5 mortality rate and summary statistics (rate and SD) by DHS cluster and district/subdistrict. Areas with too few observations of child death to create stable rates are excluded from analysis.

Partner Mapping Data

Ministry of Health and WHO officials and implementing partners in each country were asked to provide a listing, by lowest level of geography possible, the activities of implementing partners, spending, and timing of activities. We were able to obtain at least the locations of implementing partners for Ethiopia. We note two challenges to collecting and using these data:

1. For the most part, only a simple listing of partners by region was obtained. Additional information on the types of activities and funding amount for partners

was often unavailable, though would be very important for understanding the roles of these organizations. The simple presence of partners in a region (which we map here) does not necessarily mean they are providing comprehensive health system strengthening support.
2. Geographically detailed information on the location of partners is challenging to obtain. For some of the partners, zone-level information available while for others we only obtained regional activities. It is unlikely that partners are providing services in an entire region and more detailed data is necessary for understanding the impact of programs.

In addition, officials in Ethiopia provided data on the number of health centers (HCs), health posts (HPs) and health education workers (HEWs) operating in each district. The per capita number of HCs, HPs, and HEWs were calculated by dividing by the total population in each region. A limitation of these data is that only some partners reported these data, so there are many areas with no data in the maps. In other areas, the maps may underreport the true number of HCs, HPs, and HEWs.

Statistical Analysis and Mapping

The Standard DHS and Geographic Datasets were downloaded from the DHS website after providing a short description of this project and obtaining permission from the DHS Program. Data were processed in the SAS statistical software package v.9.4 to develop the health and mortality measures described above. Survey weights were applied to calculate numerators, denominators and rates in order for sample data to accurately represent the entire population. The DHS also contains data for distinct geographic areas called clusters. Clusters are groups of census enumeration areas that are used in the sampling frame to draw a geographically representative sample of the population. Survey weighted frequencies for numerators and denominators were tabulated at the cluster level and used to calculate rates and proportions. These summary measures by DHS cluster were output for use in the Geographic Information System (GIS).

Measures were linked to DHS cluster locations and mapped in the GIS using the ArcGIS v.10.1 software. The cluster locations are represented as points with a specific latitude/longitude coordinate, though this point represents the centerpoint of multiple census enumeration areas. These points were used to create a surface through a process called spatial interpolation. Interpolation is the process of obtaining a value at an unsampled location based on surrounding measurements. These smoothed maps are descriptive in nature, and are meant as a tool for visually communicate spatial patterns. There are some limitations to this method:

1. The data in the interpolated areas should not be taken as an absolute predication of the rates or prevalence in that area.
2. In areas where rates and proportions are based off small numbers and cluster points are far apart from each other (e.g., rural areas) maps can be misleading. A very high or low value at a point (driven by small numbers), is used to “fill in” data for surrounding areas with no sampled data, which can perpetuate small numbers issues and lead to large mapped areas with high/low rates.

The location of implementing partners was mapped by translating tables listing the name of the implementing partner by district/subdistrict into geographic files in the GIS. Each implementing partner was assigned a different symbol so that all partners operating in a country could be visualized simultaneously.

References

1. DHS Spatial Interpolation Working Group. 2014. *Spatial Interpolation with Demographic and Health Survey Data: Key Considerations*. DHS Spatial Analysis Reports No. 9. Rockville, MD, USA: ICF International.
2. Atwood, S. 2012. DHS_U5M: A flexible SAS macro to calculate childhood mortality estimates and standard errors from birth histories. http://hdl.handle.net/1902.1/18296. Harvard Dataverse, v1.
