# Supplementary material for: Future directions for reducing inequity and maximising impact of child health strategies
Source: BMJ. 2018 Jul 30;362:k2684. doi: 10.1136/bmj.k2684 (PMC6283368; doi:10.1136/bmj.k2684)
Supplement: Supplementary file 2 — Supplementary materials on IMCI and iCCM implementation analyses [file dals042256.ww2.pdf]

Supplementary Materials on IMCI and iCCM implementation analyses

Data

This analysis combines data from the IMCI and iCCM implementation survey, whose methodology has been described elsewhere,[1] with data from the Demographic Health Surveys (DHS), a population-representative household survey implemented by ICF International, and funded by USAID (http://www.dhsprogram.com). Further information about the DHS is provided in the Supplementary Materials describing the analyses underpinning **Figure 2** of this article.

Data from the IMCI survey were re-coded for the purposes of this analysis, with IMCI "top implementers" defined as countries in which two conditions were fulfilled: a) >90% of districts were reported to be implementing IMCI, b) all three components of the IMCI strategy were reported to be implemented. We were unable to take into account the start of implementation of either strategy within countries, and implementation was measured as a binary variable (yes/no).

The number of surveys used in the calculations are as follows:

	**Care-seeking for pneumonia**	**Care-seeking for all childhood diseases**
**IMCI**		
Time period covered	*1993-2014*	*1993-2014*
"Top implementers"	110 surveys in 31 countries	90 surveys in 31 countries
Other countries	147 surveys in 48 countries	92 surveys in 32 countries
**iCCM**		
Time period covered	*2010-2014*	*N/A*
<50% of districts implementing	27 surveys in 22 countries	N/A
≥50% of districts implementing	24 surveys in 18 countries	N/A

The selection of countries included in these analyses was based on data availability, and thus were not chosen based on their representativeness of all countries implementing IMCI. Analyses include countries from low- and middle-income countries, however not all regions are equally well-represented (e.g. while many African and Latin American countries are included, some analyses include a smaller number of Asian countries). A full list of countries is included in a separate Excel file, also under Supplementary Materials. Data is available from approximately half of the 33 fragile/conflict states listed by the World Bank[2]; while these contexts reflect particular implementation dynamics, we did not have sufficient data to analyse them separately. An inadequate focus on fragile/conflict states was noted as an overall limitation of the data collection and analysis performed under the Strategic Review.[3]

Statistical analyses

Data from the IMCI survey were cleaned and checked for errors, before being re-coded and compiled into an Excel spreadsheet. DHS datasets were downloaded from the DHS website after providing a short description of this project and obtaining permission from the DHS Program. Data were processed using the STATA statistical program, version 13.

Calculation of care-seeking rates for pneumonia and for any condition (includes pneumonia, fever and diarrhea) were made in accordance with the standard definitions used by the International Center for Equity in Health[4] and the Countdown to 2030 (www.countdown2030.org). We used multilevel linear regression models with surveys as the level one units and countries as level two units in order to estimate the annual percentage-point change in care-seeking for pneumonia and for any condition. Analyses were conducted at the global level and by wealth quintile for each category of countries according to IMCI and iCCM implementation.

References

1. Boschi-Pinto C, Labadie G, Ramachandran Thandassery D, et al. Global implementation survey of Integrated Management of Childhood Illness (IMCI)—twenty years on. *BMJ Open* 2018;8:e019079

2. World Bank. Harmonized List of Fragile Situations. Washington, DC: World Bank, 2015. http://siteresources.worldbank.org/EXTLICUS/Resources/511777-1269623894864/FY15FragileSituationList.pdf.

3. Costello A, Peterson S, Rasanathan K, Daelmans B. [Final details to be added later]. 2018.

4. Pelotas: International Centre for Equity in Health. Database for national surveys on maternal and child health in low- and middle-income countries 2016: Available from: http://www.equidade.org. Accessed on: Jul 4, 2017.
