# Supplementary material for: Future directions for reducing inequity and maximising impact of child health strategies
Source: BMJ. 2018 Jul 30;362:k2684. doi: 10.1136/bmj.k2684 (PMC6283368; doi:10.1136/bmj.k2684)
Supplement: Supplementary file 3 — Supplementary materials on care seeking for pneumonia or any disease [file dals042256.ww3.pdf]

IMCI care-seeking for pneumonia

			Survey 1		Survey 2		Survey 3		Survey 4		Survey 5		Survey 6		Survey 7		Survey 8		Survey 9		Survey 10		Survey 11	
ISO	Country	Level of implementation	Year	Source	Year	Source	Year	Source	Year	Source	Year	Source	Year	Source	Year	Source	Year	Source	Year	Source	Year	Source	Year	Source
ALB	Albania	Other	2005	MICS	2008	DHS																		
ARM	Armenia	Other	2000	DHS	2005	DHS	2010	DHS																
BDI	Burundi	High implementation in 3 components	2005	MICS	2010	DHS																		
BEN	Benin	Other	1996	DHS	2001	DHS	2006	DHS	2011	DHS														
BFA	Burkina Faso	High implementation in 3 components	1998	DHS	2003	DHS	2006	MICS	2010	DHS														
BGD	Bangladesh	High implementation in 3 components	1993	DHS	1996	DHS	1999	DHS	2004	DHS	2006	MICS	2007	DHS	2011	DHS	2012	MICS						
BIH	Bosnia and Herzegovina	Other	2006	MICS	2011	MICS																		
BLR	Belarus	Other	2005	MICS	2012	MICS																		
BLZ	Belize	Other	2006	MICS	2011	MICS																		
BOL	Bolivia	High implementation in 3 components	1994	DHS	1998	DHS	2003	DHS	2008	DHS														
BRA	Brazil	High implementation in 3 components	1996	DHS	2006	PNDS																		
CAF	CAR	Other	1994	DHS	2006	MICS	2010	MICS																
CIV	Cote dIvoire	Other	1994	DHS	1998	DHS	2006	MICS	2011	DHS														
CMR	Cameroon	Other	1998	DHS	2004	DHS	2006	MICS	2011	DHS														
COD	Congo Democratic Republic	Other	2007	DHS	2010	MICS	2013	DHS																
COG	Congo Brazzaville	High implementation in 3 components	2005	DHS	2011	DHS																		
COL	Colombia	Other	1995	DHS	2000	DHS	2005	DHS	2010	DHS														
COM	Comoros	High implementation in 3 components	1996	DHS	2012	DHS																		
CUB	Cuba	Other	2010	MICS	2014	MICS																		
DOM	Dominican Republic	Other	1996	DHS	1999	DHS	2002	DHS	2007	DHS	2013	DHS												
EGY	Egypt	High implementation in 3 components	1995	DHS	2000	DHS	2005	DHS	2008	DHS	2014	DHS												
ETH	Ethiopia	High implementation in 3 components	2000	DHS	2005	DHS	2011	DHS																
GAB	Gabon	Other	2000	DHS	2012	DHS																		
GHA	Ghana	Other	1993	DHS	1998	DHS	2003	DHS	2006	MICS	2008	DHS	2011	MICS	2014	DHS								
GIN	Guinea	Other	1999	DHS	2005	DHS	2012	DHS																
GMB	Gambia	Other	2005	MICS	2013	DHS																		
GTM	Guatemala	High implementation in 3 components	1995	DHS	1998	DHS	2008	RHS																
GUY	Guyana	High implementation in 3 components	2006	MICS	2009	DHS																		
HND	Honduras	Other	2005	DHS	2011	DHS																		
HTI	Haiti	High implementation in 3 components	1994	DHS	2000	DHS	2005	DHS	2012	DHS														
IDN	Indonesia	High implementation in 3 components	1994	DHS	1997	DHS	2002	DHS	2007	DHS	2012	DHS												
IND	India	Other	1998	DHS	2005	DHS																		
IRQ	Iraq	Other	2006	MICS	2011	MICS																		
JAM	Jamaica	Other	2005	MICS	2011	MICS																		
JOR	Jordan	Other	1997	DHS	2002	DHS	2007	DHS	2012	DHS														
KAZ	Kazakhstan	High implementation in 3 components	1995	DHS	1999	DHS	2006	MICS	2010	MICS														
KEN	Kenya	High implementation in 3 components	1993	DHS	1998	DHS	2003	DHS	2008	DHS														
KGZ	Kyrgyzstan	Other	1997	DHS	2005	MICS	2012	DHS	2014	MICS														
KHM	Cambodia	High implementation in 3 components	2000	DHS	2005	DHS	2010	DHS	2014	DHS														
LAO	Lao	Other	2006	MICS	2011	MICS																		
LBR	Liberia	High implementation in 3 components	2007	DHS	2013	DHS																		
LSO	Lesotho	Other	2004	DHS	2009	DHS																		
MDA	Moldova	High implementation in 3 components	2005	DHS	2012	MICS																		
MDG	Madagascar	Other	1997	DHS	2003	DHS	2008	DHS																
MKD	Macedonia	Other	2005	MICS	2011	MICS																		
MLI	Mali	Other	1995	DHS	2001	DHS	2006	DHS	2012	DHS														
MNE	Montenegro	Other	2005	MICS	2013	MICS																		
MNG	Mongolia	Other	2005	MICS	2010	MICS																		
MOZ	Mozambique	High implementation in 3 components	1997	DHS	2003	DHS	2008	MICS	2011	DHS														
MRT	Mauritania	Other	2007	MICS	2011	MICS																		
MWI	Malawi	High implementation in 3 components	2000	DHS	2004	DHS	2006	MICS	2010	DHS	2013	MICS												
NAM	Namibia	High implementation in 3 components	2000	DHS	2006	DHS	2013	DHS																
NER	Niger	High implementation in 3 components	1998	DHS	2006	DHS	2012	DHS																
NGA	Nigeria	Other	1999	DHS	2003	DHS	2007	MICS	2008	DHS	2011	MICS	2013	DHS										
NIC	Nicaragua	High implementation in 3 components	1998	DHS	2001	DHS																		
NPL	Nepal	Other	1996	DHS	2001	DHS	2006	DHS	2011	DHS	2014	MICS												
PAK	Pakistan	Other	2006	DHS	2012	DHS																		
PER	Peru	Other	1996	DHS	2000	DHS	2004	DHS	2005	DHS	2006	DHS	2007	DHS	2008	DHS	2009	DHS	2010	DHS	2011	DHS	2012	DHS
PHL	Philippines	Other	1993	DHS	1998	DHS	2003	DHS	2008	DHS	2013	DHS												
PIL	Palestinians in Lebanon	Other	2006	MICS	2011	MICS																		
PSE	State of Palestine	Other	2010	MICS	2014	MICS																		
RWA	Rwanda	High implementation in 3 components	2000	DHS	2005	DHS	2010	DHS																
SEN	Senegal	High implementation in 3 components	2005	DHS	2010	DHS	2012	DHS	2014	DHS														
SLE	Sierra Leone	High implementation in 3 components	2005	MICS	2008	DHS	2010	MICS	2013	DHS														
SRB	Serbia	Other	2005	MICS	2010	MICS																		
SUR	Suriname	Other	2006	MICS	2010	MICS																		
SWZ	Swaziland	Other	2006	DHS	2010	MICS																		
TCD	Chad	Other	1996	DHS	2004	DHS	2010	MICS																
TGO	Togo	Other	1998	DHS	2006	MICS	2010	MICS	2013	DHS														
TJK	Tajikistan	High implementation in 3 components	2005	MICS	2012	DHS																		
TUR	Turkey	Other	1993	DHS	2003	DHS																		
TZA	Tanzania	High implementation in 3 components	1996	DHS	1999	DHS	2004	DHS	2010	DHS														
UGA	Uganda	High implementation in 3 components	1995	DHS	2000	DHS	2006	DHS	2011	DHS														
UKR	Ukraine	Other	2012	MICS																				
UZB	Uzbekistan	Other	1996	DHS	2006	MICS																		
VNM	Vietnam	Other	1997	DHS	2002	DHS	2006	MICS	2010	MICS	2013	MICS												
YEM	Yemen	Other	2006	MICS	2013	DHS																		
ZMB	Zambia	High implementation in 3 components	1996	DHS	2001	DHS	2007	DHS	2013	DHS														
ZWE	Zimbabwe	High implementation in 3 components	1994	DHS	1999	DHS	2005	DHS	2009	MICS	2010	DHS	2014	MICS										

IMCI care-seeking for any disease

			Survey 1		Survey 2		Survey 3		Survey 4		Survey 5		Survey 6		Survey 7		Survey 8		Survey 9		Survey 10		Survey 11	
ISO	Country	Level of implementation	Year	Source	Year	Source	Year	Source	Year	Source	Year	Source	Year	Source	Year	Source	Year	Source	Year	Source	Year	Source	Year	Source
ALB	Albania	Other	2008	DHS																				
ARM	Armenia	Other	2000	DHS	2005	DHS	2010	DHS																
BDI	Burundi	High implementation in 3 components	2010	DHS																				
BEN	Benin	Other	1996	DHS	2001	DHS	2006	DHS	2011	DHS														
BFA	Burkina Faso	High implementation in 3 components	1998	DHS	2003	DHS	2010	DHS																
BGD	Bangladesh	High implementation in 3 components	1996	DHS	1999	DHS	2004	DHS	2007	DHS	2011	DHS												
BOL	Bolivia	High implementation in 3 components	1994	DHS	1998	DHS	2003	DHS																
BRA	Brazil	High implementation in 3 components	1996	DHS																				
CAF	CAR	Other	1994	DHS																				
CIV	Cote_dIvoire	Other	1994	DHS	1998	DHS	2011	DHS																
CMR	Cameroon	Other	1998	DHS	2004	DHS	2011	DHS																
COD	Congo_Democratic_Republic	Other	2007	DHS	2013	DHS																		
COG	Congo_Brazzaville	High implementation in 3 components	2005	DHS	2011	DHS																		
COL	Colombia	Other	1995	DHS	2000	DHS	2005	DHS	2010	DHS														
COM	Comoros	High implementation in 3 components	1996	DHS	2012	DHS																		
DOM	Dominican_Republic	Other	1996	DHS	1999	DHS	2002	DHS	2007	DHS	2013	DHS												
EGY	Egypt	High implementation in 3 components	1995	DHS	2000	DHS	2005	DHS	2008	DHS	2014	DHS												
ETH	Ethiopia	High implementation in 3 components	2000	DHS	2005	DHS	2011	DHS																
GAB	Gabon	Other	2000	DHS	2012	DHS																		
GHA	Ghana	Other	1993	DHS	1998	DHS	2003	DHS	2008	DHS	2014	DHS												
GIN	Guinea	Other	1999	DHS	2005	DHS	2012	DHS																
GMB	Gambia	Other	2013	DHS																				
GTM	Guatemala	High implementation in 3 components	1995	DHS	1998	DHS																		
GUY	Guyana	High implementation in 3 components	2009	DHS																				
HND	Honduras	Other	2005	DHS	2011	DHS																		
HTI	Haiti	High implementation in 3 components	1994	DHS	2000	DHS	2005	DHS	2012	DHS														
IDN	Indonesia	High implementation in 3 components	1994	DHS	1997	DHS	2002	DHS	2007	DHS	2012	DHS												
IND	India	Other	1998	DHS	2005	DHS																		
JOR	Jordan	Other	1997	DHS	2002	DHS	2007	DHS	2012	DHS														
KAZ	Kazakhstan	High implementation in 3 components	1995	DHS	1999	DHS																		
KEN	Kenya	High implementation in 3 components	1993	DHS	1998	DHS	2003	DHS	2008	DHS														
KGZ	Kyrgyzstan	Other	1997	DHS	2012	DHS																		
KHM	Cambodia	High implementation in 3 components	2000	DHS	2005	DHS	2010	DHS	2014	DHS														
LBR	Liberia	High implementation in 3 components	2007	DHS	2013	DHS																		
LSO	Lesotho	Other	2004	DHS	2009	DHS																		
MDA	Moldova	High implementation in 3 components	2005	DHS																				
MDG	Madagascar	Other	1997	DHS	2003	DHS	2008	DHS																
MLI	Mali	Other	1995	DHS	2001	DHS	2006	DHS	2012	DHS														
MOZ	Mozambique	High implementation in 3 components	1997	DHS	2003	DHS	2011	DHS																
MWI	Malawi	High implementation in 3 components	2000	DHS	2004	DHS	2010	DHS																
NAM	Namibia	High implementation in 3 components	2000	DHS	2006	DHS	2013	DHS																
NER	Niger	High implementation in 3 components	1998	DHS	2006	DHS	2012	DHS																
NGA	Nigeria	Other	1999	DHS	2003	DHS	2008	DHS	2013	DHS														
NIC	Nicaragua	High implementation in 3 components	1998	DHS	2001	DHS																		
NPL	Nepal	Other	1996	DHS	2001	DHS	2006	DHS	2011	DHS														
PAK	Pakistan	Other	2006	DHS	2012	DHS																		
PER	Peru	Other	1996	DHS	2000	DHS	2004	DHS	2005	DHS	2006	DHS	2007	DHS	2008	DHS	2009	DHS	2010	DHS	2011	DHS	2012	DHS
PHL	Philippines	Other	1993	DHS	1998	DHS	2003	DHS	2008	DHS	2013	DHS												
RWA	Rwanda	High implementation in 3 components	2000	DHS	2005	DHS	2010	DHS																
SEN	Senegal	High implementation in 3 components	2005	DHS	2010	DHS	2012	DHS	2014	DHS														
SLE	Sierra_Leone	High implementation in 3 components	2008	DHS	2013	DHS																		
SWZ	Swaziland	Other	2006	DHS																				
TCD	Chad	Other	1996	DHS	2004	DHS																		
TGO	Togo	Other	1998	DHS	2013	DHS																		
TJK	Tajikistan	High implementation in 3 components	2012	DHS																				
TUR	Turkey	Other	1993	DHS																				
TZA	Tanzania	High implementation in 3 components	1996	DHS	1999	DHS	2004	DHS	2010	DHS														
UGA	Uganda	High implementation in 3 components	1995	DHS	2000	DHS	2006	DHS	2011	DHS														
UZB	Uzbekistan	Other	1996	DHS																				
VNM	Vietnam	Other	1997	DHS	2002	DHS																		
YEM	Yemen	Other	2013	DHS																				
ZMB	Zambia	High implementation in 3 components	1996	DHS	2001	DHS	2007	DHS	2013	DHS														
ZWE	Zimbabwe	High implementation in 3 components	1994	DHS	1999	DHS	2005	DHS	2010	DHS														

iCCM care-seeking for pneumonia

			Survey 1		Survey 2	
ISO	Country	Level of implementation	Year	Source	Year	Source
ARM	Armenia	>=50%	2010	DHS		
BDI	Burundi	<50%	2010	DHS		
BEN	Benin	>=50%	2011	DHS		
BFA	Burkina_Faso	>=50%	2010	DHS		
BGD	Bangladesh	>=50%	2011	DHS	2012	MICS
CIV	Cote_dIvoire	>=50%	2011	DHS		
CMR	Cameroon	<50%	2011	DHS		
COD	Congo_Democratic_Republic	>=50%	2010	MICS	2013	DHS
COG	Congo_Brazzaville	<50%	2011	DHS		
COL	Colombia	<50%	2010	DHS		
COM	Comoros	<50%	2012	DHS		
CUB	Cuba	>=50%	2010	MICS	2014	MICS
DOM	Dominican_Republic	<50%	2013	DHS		
EGY	Egypt	<50%	2014	DHS		
ETH	Ethiopia	>=50%	2011	DHS		
GHA	Ghana	>=50%	2011	MICS	2014	DHS
GIN	Guinea	>=50%	2012	DHS		
GMB	Gambia	>=50%	2013	DHS		
HTI	Haiti	<50%	2012	DHS		
IDN	Indonesia	<50%	2012	DHS		
KHM	Cambodia	<50%	2010	DHS	2014	DHS
LAO	Lao	<50%	2011	MICS		
LBR	Liberia	<50%	2013	DHS		
MLI	Mali	>=50%	2012	DHS		
MOZ	Mozambique	>=50%	2011	DHS		
MWI	Malawi	>=50%	2010	DHS	2013	MICS
NER	Niger	>=50%	2012	DHS		
NGA	Nigeria	<50%	2011	MICS	2013	DHS
NPL	Nepal	>=50%	2011	DHS	2014	MICS
PAK	Pakistan	<50%	2012	DHS		
PHL	Philippines	<50%	2013	DHS		
RWA	Rwanda	>=50%	2010	DHS		
SLE	Sierra_Leone	<50%	2010	MICS	2013	DHS
TCD	Chad	<50%	2010	MICS		
TGO	Togo	<50%	2010	MICS	2013	DHS
TJK	Tajikistan	>=50%	2012	DHS		
UGA	Uganda	<50%	2011	DHS		
YEM	Yemen	<50%	2013	DHS		
ZMB	Zambia	<50%	2013	DHS		
ZWE	Zimbabwe	<50%	2010	DHS	2014	MICS

Fragile states - IMCI care-seeking for pneumonia

ISO	Country	Level of implementation	Fragile state?
ALB	Albania	Other	NO
ARM	Armenia	Other	NO
BDI	Burundi	High implementation in 3 components	YES
BEN	Benin	Other	NO
BFA	Burkina_Faso	High implementation in 3 components	NO
BGD	Bangladesh	High implementation in 3 components	NO
BIH	Bosnia_and_Herzegovina	Other	YES
BLR	Belarus	Other	NO
BLZ	Belize	Other	NO
BOL	Bolivia	High implementation in 3 components	NO
BRA	Brazil	High implementation in 3 components	NO
CAF	CAR	Other	YES
CIV	Cote_dIvoire	Other	NO
CMR	Cameroon	Other	NO
COD	Congo_Democratic_Republic	Other	YES
COG	Congo_Brazzaville	High implementation in 3 components	NO
COL	Colombia	Other	NO
COM	Comoros	High implementation in 3 components	YES
CUB	Cuba	Other	NO
DOM	Dominican_Republic	Other	NO
EGY	Egypt	High implementation in 3 components	NO
ETH	Ethiopia	High implementation in 3 components	NO
GAB	Gabon	Other	NO
GHA	Ghana	Other	NO
GIN	Guinea	Other	NO
GMB	Gambia	Other	NO
GTM	Guatemala	High implementation in 3 components	NO
GUY	Guyana	High implementation in 3 components	NO
HND	Honduras	Other	NO
HTI	Haiti	High implementation in 3 components	YES
IDN	Indonesia	High implementation in 3 components	NO
IND	India	Other	NO
IRQ	Iraq	Other	YES
JAM	Jamaica	Other	NO
JOR	Jordan	Other	NO
KAZ	Kazakhstan	High implementation in 3 components	NO
KEN	Kenya	High implementation in 3 components	NO
KGZ	Kyrgyzstan	Other	NO
KHM	Cambodia	High implementation in 3 components	NO
LAO	Lao	Other	NO
LBR	Liberia	High implementation in 3 components	YES
LSO	Lesotho	Other	NO
MDA	Moldova	High implementation in 3 components	NO
MDG	Madagascar	Other	YES
MKD	Macedonia	Other	NO
MLI	Mali	Other	YES
MNE	Montenegro	Other	NO
MNG	Mongolia	Other	NO
MOZ	Mozambique	High implementation in 3 components	NO
MRT	Mauritania	Other	NO
MWI	Malawi	High implementation in 3 components	NO
NAM	Namibia	High implementation in 3 components	NO
NER	Niger	High implementation in 3 components	NO
NGA	Nigeria	Other	NO
NIC	Nicaragua	High implementation in 3 components	NO
NPL	Nepal	Other	NO
PAK	Pakistan	Other	NO
PER	Peru	Other	NO
PHL	Philippines	Other	NO
PIL	Palestinians_in_Lebanon	Other	NO
PSE	State_of_Palestine	Other	NO
RWA	Rwanda	High implementation in 3 components	NO
SEN	Senegal	High implementation in 3 components	NO
SLE	Sierra_Leone	High implementation in 3 components	YES
SRB	Serbia	Other	NO
SUR	Suriname	Other	NO
SWZ	Swaziland	Other	NO
TCD	Chad	Other	YES
TGO	Togo	Other	YES
TJK	Tajikistan	High implementation in 3 components	NO
TUR	Turkey	Other	NO
TZA	Tanzania	High implementation in 3 components	NO
UGA	Uganda	High implementation in 3 components	NO
UKR	Ukraine	Other	NO
UZB	Uzbekistan	Other	NO
VNM	Vietnam	Other	NO
YEM	Yemen	Other	YES
ZMB	Zambia	High implementation in 3 components	NO
ZWE	Zimbabwe	High implementation in 3 components	YES

From the 33 fragile states listed in this document: http://siteresources.worldbank.org/EXTLICUS/Resources/511777-1269623894864/FY15FragileSituationList.pdf, we have information on 15, almost a half of them. Also, from the 79 countries included, 15 are from fragile states (19%)

Fragile states - IMCI care-seeking for any disease

ISO	Country	Level of implementation	Fragile State?
ALB	Albania	Other	NO
ARM	Armenia	Other	NO
BDI	Burundi	High implementation in 3 components	YES
BEN	Benin	Other	NO
BFA	Burkina_Faso	High implementation in 3 components	NO
BGD	Bangladesh	High implementation in 3 components	NO
BOL	Bolivia	High implementation in 3 components	NO
BRA	Brazil	High implementation in 3 components	NO
CAF	CAR	Other	YES
CIV	Cote_dIvoire	Other	NO
CMR	Cameroon	Other	NO
COD	Congo_Democratic_Republic	Other	YES
COG	Congo_Brazzaville	High implementation in 3 components	NO
COL	Colombia	Other	NO
COM	Comoros	High implementation in 3 components	YES
DOM	Dominican_Republic	Other	NO
EGY	Egypt	High implementation in 3 components	NO
ETH	Ethiopia	High implementation in 3 components	NO
GAB	Gabon	Other	NO
GHA	Ghana	Other	NO
GIN	Guinea	Other	NO
GMB	Gambia	Other	NO
GTM	Guatemala	High implementation in 3 components	NO
GUY	Guyana	High implementation in 3 components	NO
HND	Honduras	Other	NO
HTI	Haiti	High implementation in 3 components	YES
IDN	Indonesia	High implementation in 3 components	NO
IND	India	Other	NO
JOR	Jordan	Other	NO
KAZ	Kazakhstan	High implementation in 3 components	NO
KEN	Kenya	High implementation in 3 components	NO
KGZ	Kyrgyzstan	Other	NO
KHM	Cambodia	High implementation in 3 components	NO
LBR	Liberia	High implementation in 3 components	YES
LSO	Lesotho	Other	NO
MDA	Moldova	High implementation in 3 components	NO
MDG	Madagascar	Other	YES
MLI	Mali	Other	YES
MOZ	Mozambique	High implementation in 3 components	NO
MWI	Malawi	High implementation in 3 components	NO
NAM	Namibia	High implementation in 3 components	NO
NER	Niger	High implementation in 3 components	NO
NGA	Nigeria	Other	NO
NIC	Nicaragua	High implementation in 3 components	NO
NPL	Nepal	Other	NO
PAK	Pakistan	Other	NO
PER	Peru	Other	NO
PHL	Philippines	Other	NO
RWA	Rwanda	High implementation in 3 components	NO
SEN	Senegal	High implementation in 3 components	NO
SLE	Sierra_Leone	High implementation in 3 components	YES
SWZ	Swaziland	Other	NO
TCD	Chad	Other	YES
TGO	Togo	Other	YES
TJK	Tajikistan	High implementation in 3 components	NO
TUR	Turkey	Other	NO
TZA	Tanzania	High implementation in 3 components	NO
UGA	Uganda	High implementation in 3 components	NO
UZB	Uzbekistan	Other	NO
VNM	Vietnam	Other	NO
YEM	Yemen	Other	YES
ZMB	Zambia	High implementation in 3 components	NO
ZWE	Zimbabwe	High implementation in 3 components	YES

From the 33 fragile states listed in this document: http://siteresources.worldbank.org/EXTLICUS/Resources/511777-1269623894864/FY15FragileSituationList.pdf, we have information on 13, almost a half of them. Also, from the 63 countries included, 13 are from fragile states (21%)

Fragile states - iCCM care-seeking for pneumonia

ISO	Country	Level of implementation	Fragile State?
ARM	Armenia	>=50%	NO
BDI	Burundi	<50%	YES
BEN	Benin	>=50%	NO
BFA	Burkina_Faso	>=50%	NO
BGD	Bangladesh	>=50%	NO
CIV	Cote_dIvoire	>=50%	NO
CMR	Cameroon	<50%	NO
COD	Congo_Democratic_Republic	>=50%	YES
COG	Congo_Brazzaville	<50%	NO
COL	Colombia	<50%	NO
COM	Comoros	<50%	YES
CUB	Cuba	>=50%	NO
DOM	Dominican_Republic	<50%	NO
EGY	Egypt	<50%	NO
ETH	Ethiopia	>=50%	NO
GHA	Ghana	>=50%	NO
GIN	Guinea	>=50%	NO
GMB	Gambia	>=50%	NO
HTI	Haiti	<50%	YES
IDN	Indonesia	<50%	NO
KHM	Cambodia	<50%	NO
LAO	Lao	<50%	NO
LBR	Liberia	<50%	YES
MLI	Mali	>=50%	YES
MOZ	Mozambique	>=50%	NO
MWI	Malawi	>=50%	YES
NER	Niger	>=50%	NO
NGA	Nigeria	<50%	NO
NPL	Nepal	>=50%	NO
PAK	Pakistan	<50%	NO
PHL	Philippines	<50%	NO
RWA	Rwanda	>=50%	NO
SLE	Sierra_Leone	<50%	YES
TCD	Chad	<50%	YES
TGO	Togo	<50%	YES
TJK	Tajikistan	>=50%	NO
UGA	Uganda	<50%	NO
YEM	Yemen	<50%	YES
ZMB	Zambia	<50%	NO
ZWE	Zimbabwe	<50%	YES

From the 33 fragile states listed in this document: http://siteresources.worldbank.org/EXTLICUS/Resources/511777-1269623894864/FY15FragileSituationList.pdf, we have information on 12, almost a half of them. Also, from the 40 countries included, 12 are from fragile states (30%)
